# Effects of non-vitamin K antagonist oral anticoagulants on fibrin clot and whole blood clot formation, integrity and thrombolysis in patients with atrial fibrillation

**DOI:** 10.1007/s11239-016-1399-3

**Published:** 2016-08-23

**Authors:** Yee Cheng Lau, Qinmei Xiong, Eduard Shantsila, Gregory Y. H. Lip, Andrew D. Blann

**Affiliations:** 1 Institute for Cardiovascular Sciences, City Hospital, University of Birmingham, Dudley Road, Birmingham, B18 7QH UK; 2 Cardiovascular Department, The Second Affiliated Hospital of Nanchang University, Nanchang, China

**Keywords:** Atrial fibrillation, Fibrinolysis, NOAC, Thrombelastograph, Thrombosis

## Abstract

Non-vitamin K antagonist oral anticoagulants (NOACs) are replacing warfarin and heparins in several clinical situations. With varying modes of action, the effects of NOACs on thrombus formation, integrity, and lysis is unknown. To determine whether two techniques of thrombelastography (TEG) and a micro-plate assay (MPA) provide novel data on thrombus formation, integrity and lysis in those taking a NOACs compared to warfarin and a control group taking aspirin. We assessed thrombogenesis, clot integrity and fibrinolysis in blood (TEG) and plasma (MPA) from 182 atrial fibrillation patients—50 on aspirin, 50 on warfarin, and 82 on a NOAC (17 apixaban, 19 dabigatran and 46 rivaroxaban). Eleven of 16 TEG indices and 4 of 5 MPA indices differed (*p* ≤ 0.01) between those on aspirin, warfarin or a NOAC. Three TEG indices and 4 MPA indices differed (*p* < 0.01) between the NOACs. Time to initiation of clot formation was most rapid on apixaban, then rivaroxaban and slowest on dabigatran. The rate of clot formation was most rapid on dabigatran, then apixaban, and slowest on rivaroxaban. Clot density was greatest on rivaroxaban, then apixaban, but weakest on dabigatran. The rate of clot dissolution was most rapid in apixaban, then dabigatran, and slowest on rivaroxaban. The TEG and MPA identify major differences in thrombogenesis and fibrinolysis in different NOACs. These techniques may have value in investigating the effects of these drugs on haemostasis in a clinical setting, and in identifying those in need of targeted therapy.

## Introduction

The introduction of non-vitamin K antagonist oral anticoagulants (NOACs) has fundamentally changed clinical practice in the use of oral and parenteral anticoagulation therapy in the prevention of stroke in atrial fibrillation (AF), and in the prevention and treatment of venous thromboembolism, including after orthopaedic surgery. Unlike vitamin K antagonists (VKAs, such as warfarin), which non-specifically suppress hepatic production of functional coagulation factors II, VII, IX and X by inhibiting vitamin K metabolism, NOACs target specific molecules of the coagulation cascade: dabigatran is a direct thrombin inhibitor whilst apixaban and rivaroxaban both target factor Xa [1–4].

NOACs have several advantages over VKAs, one being that their predictable pharmacokinetics allows for elimination of routine anticoagulation monitoring. However, there are a small number of instances, such as in emergency surgery and in suspected overdose, where precise knowledge of the degree of anticoagulation is called for [4, 5]. Although NOACs may in exceptional (emergency) circumstances be assessed by standard laboratory tests (such as the prothrombin time and the activated partial thromboplastin time), lack of sensitivity and specificity of these tests calls for other assays (such as ecarin clotting time and anti-Factor Xa activity) [4–6]. A further feature of NOACs use is that renal function must be assessed as use of these drugs require caution in those with severe renal failure [7].

Other laboratory tools for determining the rate of thrombus formation and fibrinolysis in whole blood and plasma include techniques such as the thrombelastograph (TEG) [8] and an assay performed in a 96-well microtitre plate, of which there are several variants [9–11]. The TEG delivers numerous indices, such as the time for coagulation to begin, the rate of clot formation, the physical strength of the clot, and the rate of auto-fibrinolysis, and has been used to manage patients experiencing severe bleeding resulting from the polytrauma of cardiac surgery, in renal disease, and to compare anticoagulants [12–15]. Our recently described microplate assay (MPA) [9] provides five similar measures, including the time for coagulation to begin and the rate of clot formation, but also on the density of the fibrin clot and its resistance to fibrinolysis by exogenous tissue plasminogen activator (tPA). The effect of the clinical use of NOACs on these two techniques has yet to be determined, and may provide novel perspectives on the action of these drugs.

Therefore, we hypothesized that the TEG and MPA methods will demonstrate difference in indices of clot formation, integrity and lysis between blood and plasma from those taking warfarin and the three commonly used NOACs. We used AF as a model in which to test these hypotheses, as in different clinical situations patients may be on warfarin or on a NOAC. Furthermore, as AF patients may be taking aspirin, these provide a control group of blood and plasma where the coagulation pathway is essentially undisturbed.

## Patients and methods

### Subjects

Consecutive patients with AF were recruited from routine out-patient cardiology clinics, and all had been taking their anti-thrombotic for at least 4 weeks. Doses were according to UK guidelines, e.g. apixaban 5 mg bd, dabigatran 150 mg bd, rivaroxaban 20 mg od, warfarin titrated to achieve an international normalised ratio (INR) between 2.0 and 3.0. For those on warfarin, INR was determined on the day of testing to assess effective anticoagulation. For those receiving NOACs, venepuncture took place 4–6 h after the daily dose of the drug. Exclusion criteria were age <18 years, active or recent (<12 months) malignancy, active immunological disease, pregnancy, chronic liver disease, recent or chronic infections, chronic inflammatory disease, connective tissue disease, recent stroke/acute coronary syndrome (within 2 months), active bleeding, recent arterial/venous thrombosis or recent surgery, known haemophilia or thrombophilia (such as Factor V Leiden, Protein C/S/anti-thrombin deficiency, anti-phospholipid syndrome), use of an anti-platelet agent other than aspirin, use of a VKA other than warfarin, and dual anti-thrombotic therapy. Standard clinical and demographic data were obtained, and a routine blood test was taken for renal function. The project has been approved by the Local Research Ethics Committee and informed consent was obtained from each participant.

### Laboratory

**Fig. 1:**
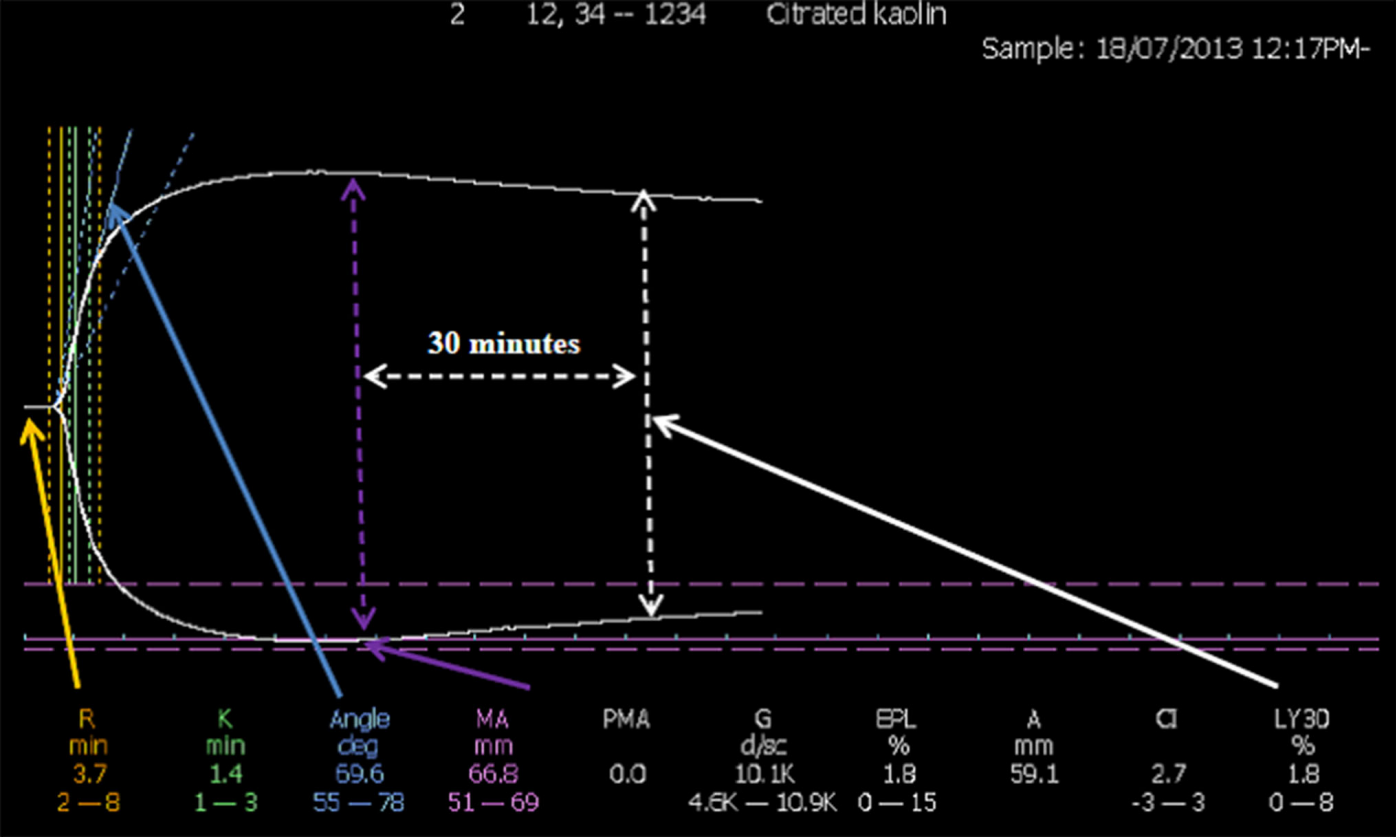
A typical TEG printout. Typical TEG graphical tracing: amplitude against time. *A* Amplitude, *CI* coagulation index, *EPL* estimated potential lysis, *G* G-parameter, *K* K-time, *LY30* percentage of lysis 30 min post maximum amplitude is attained, *MA* maximum amplitude, *PMA* projected maximum amplitude, *R* R-time. Figures are manufacturer’s reference ranges

**Fig. 2:**
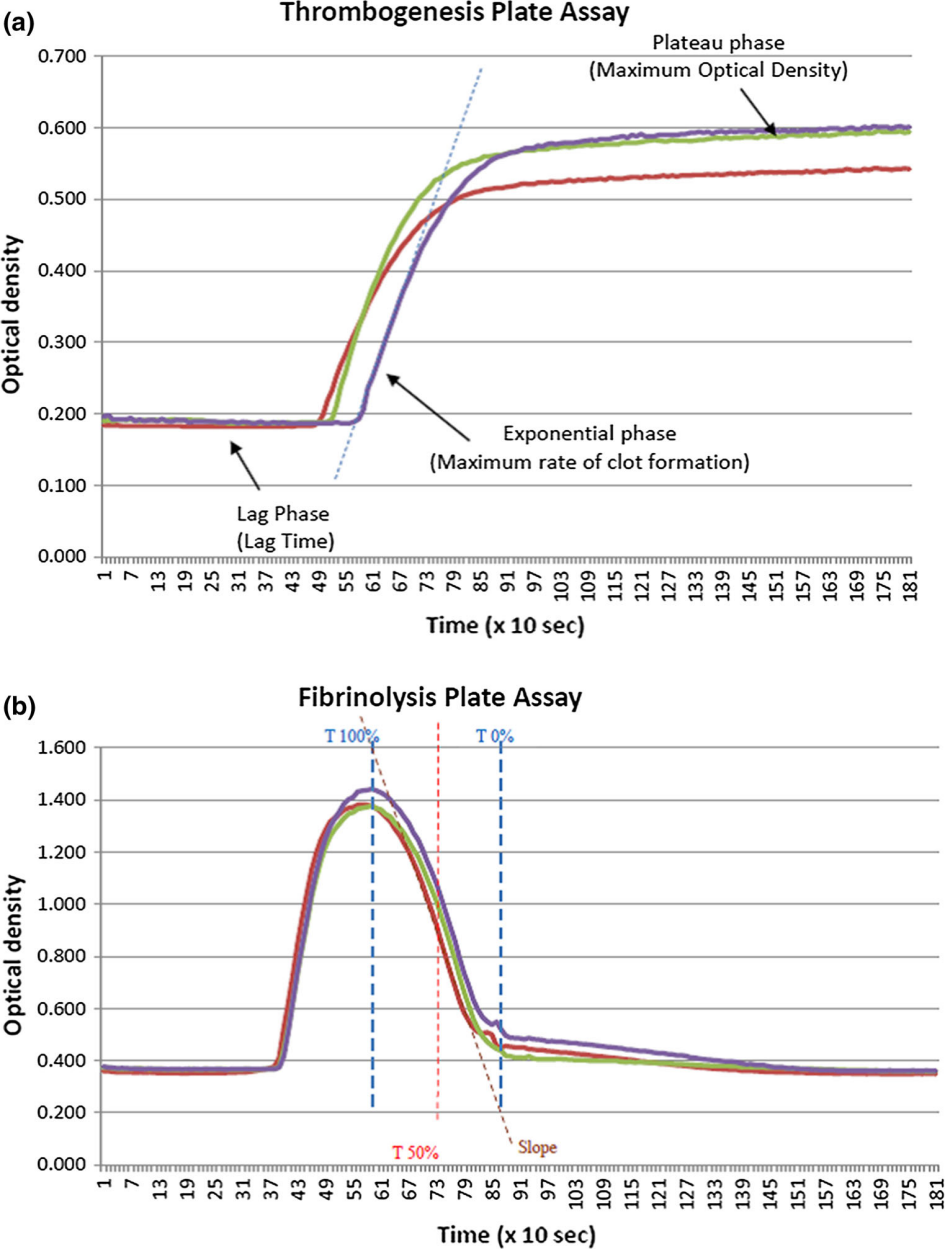
Data from the MPA. **a** Thrombogenesis. The plot shows changes in optical density as the fibrin clot forms follwing initiation (time = 0 s). Triplicate plots are shown. **b** Fibrinolysis. The plot shows changes in optical density as the fibrin clot forms. Triplicate plots are shown. T100 % is the time to maximum absorbance, T0 % is the return of the optical density to near-baseline. T50 % is (T100 %—T0 %)/2. The slope is the sharpest fall in optical density over time under the effect of exogenous tPA, effectively the reverse of the rate of clot formation in **a**

Citrated venous blood was collected by venepuncture and analysed for indices of thrombogenesis and fibrinolysis within 2 h of collection. The TEG was used according to the manufacturer’s instructions. Thrombus formation and autolysis were monitored for up to 60 min after the addition of calcium to whole blood supplemented with kaolin, in which cases it is methodologically allied to the activated partial thromboplastin time (Fig. 1). Table 1 describes 16 of the most appropriate TEG indices that can be summarised as those pertaining to the initiation of thrombosis, the rate of thrombus formation, the physical strength of the clot once formed, and the rate of autolysis. For the MPA, citrated plasma was obtained from venous sample by centrifugation at 2500 rpm for 15 min [9]. The MPA, which is conducted at 37 °C throughout, consists of two parts. Firstly, in a thrombogenesis assay, 25 μl plasma, 75 μl TRIS-NaCl buffer, and 50 μl thrombin/calcium are added to the wells of 96-well microtitre plate in triplicate (final concentration of thrombin in the reaction vessel = 0.473 IU/ml). Clot formation is followed for 30 min in a vibrating micro-titre plate reader by changes in optical density (Fig. 2a). Secondly, the fibrinolysis assay calls for 75 µL of plasma and 75 µL of a Tris/NaCl/calcium buffer supplemented with thrombin and tPA (final concentration of tPA in the reaction vessel = 10 μg/ml). The plate is immediately loaded into the plate reader as for the thrombogenesis assay, and data collected for 30 min. The data is post-processed to plot into line charts, and from these the rate of clot dissolution (RCD) and the time for 50 % of the clot to lyse (T50) are calculated. The RCD is defined as the fall in optical density from its maximum (peak) value to the elbow point, divided by the time between these two points. The T50 is defined as the half of the time interval between the peak and the elbow point (Fig. 2b).

**Table 1:** TEG and MPA indices

	Process assessing
TEG indices	
R time	Time from the initiation of the test until the point where the clot begins to form
K time	Interval from the split point of the test to the point where the fibrin cross-linking provides enough clot resistance to produce a 20-mm amplitude
Angle	Angle formed by the slope of a tangent line traced from the R time to the K time: reflects the rate at which the clot forms
MA	Maximum amplitude of the clot dynamics, reflecting clot strength
TMA	Time to maximum amplitude (when the clot is strongest)
G	Shear-elastic modulus of the strength of the clot
E	Normalised G parameter
TPI	Thrombo-dynamic potential index: The elastic shear modulus divided by kinetics of clot development, reflecting global coagulation
A30	Amplitude (reflecting clot strength) after 30 min
CL30	Proportion of the clot (as a percentage) that remains unlysed after 30 min
LY30	Percentage of the clot that has lysed 30 min after the time of MA
A60	Amplitude (reflecting clot strength) after 60 min
CL60	Proportion of the clot (as a percentage) that remains unlysed lysed after 60 min
LY60	Percentage of the clot that has lysed 60 min after the time of MA
A	Amplitude: measures the width of tracing at the latest time point
CI	Coagulation index: assessment of overall coagulation derived from other indices including the R time, K time, MA and the angle
MPA indices	
L time	Lag time from the initiation of the test to the start of clot formation
RCF	Rate of clot formation: change in optical density over time from the beginning of clot formation to maximum optical density
MOD	Maximum optical density, reflecting clot thickness
RCD	Rate of clot dissolution: reduction in optical density from maximum to the plateau phase
T50	Time for 50 % of the clot to lyse

TEG definitions as provided by manufacturer. Full details of MPA indices in Ref. [9]. See also Figs. 1 and 2

The five indices produced by the MPA also reflect clot formation, density and lysis (Table 1), and reflect several of those of the TEG. For example, the TEG R time and MPA L time both measure time taken for thrombogenesis to begin. The TEG K time marks time from the beginning of clot formation to when a fixed level of clot firmness is reached: there is no MPA equivalent of this index. The TEG angle and the MPA rate of clot formation (RCF) both measure the rate clot growth. The TEG maximum amplitude (MA) and MPA maximum optical density (MOD) respectively both assess maximum strength, stiffness or density of the developed clot. The TEG LY and CL indices, the MPA results of the RCD and the T50 all provide indices of the ability of the formed clot to resist fibrinolysis. Further details of the relationship between the TEG and the MPA are presented elsewhere [9].

### Statistical analysis

We hypothesised a difference in a continuously variable test statistic of a third of a standard deviation between any two of the major antithrombotic drug groups. For an overall analysis of variance (ANOVA) of *p* < 0.01 and *p* < 0.05 between groups at 1 = beta = 0.8, then a sample size of 50 per group is required. We recruited consecutively until this target was achieved. Upon completion, there were at least 17 patients in each NOAC group. This sample size provides the overall ANOVA of *p* < 0.02, and *p* < 0.05 between groups for a difference of half a standard deviation at 1-beta = 0.8. Continuously variable data are expressed as mean and standard deviation (SD) or median and interquartile range (IQR) dependent on distribution, and analysed by analysis of variance or the Kruskall–Wallis test. Differences between groups were sought by Tukey’s post hoc test (log transformed if necessary). Categorical indices were analysed by the Chi squared test. Correlations were sought using Spearman’s method. Analyses were performed on Minitab 16, and, in view of the multiple analyses, *p* ≤ 0.01 was considered significant.

## Results

Clinical and demographic details of the 182 participants managed by different antithrombotic agents are shown in Table 2. There were no significant differences in age, sex, blood pressure or renal function although there were proportionally more patients with ischaemic heart disease in the aspirin (34 %) and warfarin (42 %) groups compared to those on a NOAC (21 %). Patients on warfarin had a higher body mass index than those on aspirin. The mean INR was 2.6 (0.7) in those on warfarin.

**Table 2:** Clinical and demographic details

	Aspirin (*n* = 50)	Warfarin (*n* = 50)	Apixaban (*n* = 17)	Dabigatran (*n* = 19)	Rivaroxaban (*n* = 46)	*p*
Age (years)	73.3 (13.2)	73.9 (9.0)	72.1 (10.6)	76.9 (10.6)	71.6 (8.5)	0.443
Sex (male/female)	37/13	32/18	8/9	15/4	26/20	0.122
SBP (mm Hg)	130 (18)	130 (19)	135 (18)	126 (22)	132 (18)	0.641
DBP (mm Hg)	74 (13)	73 (12)	76 (14)	73 (15)	73 (11)	0.915
BMI (kg/m^2^)	26.1 (5.6)	29.6 (4.3)	27.4 (5.3)	26.7 (4.3)	30.1 (7.3)	0.004
Creatinine (µmol/l)	103 (51)	87 (15)	86 (30)	90 (22)	88 (21)	0.082
eGFR (ml/min/1.73)	64 (21)	70 (10)	68 (19)	71 (14)	69 (15)	0.265
IHD (yes/no)	17/33	21/29	3/14	6/13	8/38	0.007
Smoking (yes/no)	3/47	1/49	0/17	1/18	3/43	*
Diabetes (yes/no)	8/42	16/34	6/11	3/19	12/34	0.196
Hypertension (yes/no)	42/8	43/7	15/2	14/5	33/13	0.302
Heart failure (yes/no)	10/40	15/35	1/16	1/18	10/36	0.099
Valve disease (yes/no)	1/49	3/47	0/17	0/19	3/43	*
Pulmonary disease (yes/no)	5/45	4/46	3/14	1/18	5/41	0.762

Data presented as mean (standard deviation) or number of patients. *p* values by analysis of variance or the Chi squared test. *SBP* systolic blood pressure, *DBP* diastolic blood pressure, *BMI* body mass index, *eGFR* estimated glomerular filtration rate, *IHD* ischaemic heart disease

*Analysis unreliable

Table 3 shows age, sex, renal function, TEG indices and MPA indices in those on aspirin, warfarin or a NOAC. Patients on aspirin had higher mean serum creatinine levels than the two other groups but renal function was similar between the warfarin and NOAC groups, probably reflecting practitioners’ reluctance to place those with poor renal function on a NOAC [5, 7]. There were differences in 11 of the 16 TEG indices: 7 were between the aspirin group and the two other groups. For example, warfarin and (as a whole) NOACs both extended the TMA by slightly under 20 %. TEG indices CL30, LY30, CL60 and LY60 differed between those on warfarin and those in the combined NOAC group. These four indices inter-correlated with correlation coefficients between 0.91 and 0.99. In the 50 patients on patients on warfarin, the INR significantly correlated with TEG indices of R time (*r* = 0.47, *p* = 0.001), the TMA (*r* = 0.40, *p* = 0.004) and the CI (*r* = −0.42, *p* = 0.003). The INR correlated with MPA indices L time (*r* = 0.40, *p* = 0.004) and RCF (*r* = −0.57, *p* < 0.001).

**Table 3:** Analysis according to anti-thrombotic drug class

	Aspirin (*n* = 50)	NOAC (*n* = 82)	Warfarin (*n* = 50)	*p*
Clinical and demographic				
Age (years)	73.7 (13.2)	73.5 (10.3)	71.6 (8.5)	0.515
Sex (male/female)	37/13	49/33	32/18	0.248
Creatinine (µmol/l)	103 (51)	88 (24)	87 (15)	0.017
eGFR (ml/min/1.73)	64 (21)	69 (16)	70 (10)	0.086
TEG indices				
R (min)	4.9 (1.5)	8.6 (4.0)	7.8 (4.6)	<0.001^a^
K (min)	1.3 (1.07–1.72)	1.8 (1.47–2.2)	1.8 (1.5–2.3)	<0.001^a^
Angle (°)	69.2 (7.0)	63.1 (8.2)	60.9 (11.0)	<0.001^a^
MA (mm)	67.9 (6.1)	65.3 (7.3)	63.4 (12.8)	0.038
TMA (min)	23.9 (3.5)	28.6 (6.0)	28.5 (7.0)	<0.001^a^
G (d/s/10^3^)	11.2 (3.5)	9.9 (2.8)	9.8 (3.5)	0.042^a^
E (d/s)	224 (71)	199 (56)	195 (69)	0.042^a^
TPI (s)	93 (55)	61 (35)	60 (42)	<0.001^a^
A30 (mm)	65.8 (6.8)	63.2 (7.3)	62.3 (12.7)	<0.001^a^
CL30 (%)	96.8 (2.8)	96.9 (2.6)	98.1 (2.1)	0.01^b^
LY30	0.55 (0.2–1.3)	0.65 (0.1–1.4)	0.10 (0.0–0.60)	0.004^b^
A60 (mm)	61.7 (6.9)	59.4 (7.1)	59.2 (11.9)	0.277
CL60 (%)	90.7 (3.7)	91.0 (4.3)	93.5 (4.1)	0.001^b^
LY60 (%)	3.5 (2.4–5.1)	3.3 (1.8–5.2)	2.0 (0.9–3.5)	0.002^b^
A (mm)	61.6 (6.9)	58.9 (9.3)	59.4 (11.3)	0.248
CI (arbitrary units)	2.0 (0.47–3.4)	−0.7 (−2.7–1.1)	0 (−2.2–1.3)	<0.001^a^
MPA indices				
L time (min)	5.5 (4.8–6.0)	9.6 (7.8–13.0)	8.3 (6.8–9.5)	<0.001^a^
RCF (units/s)	39.8 (36.5–44.3)	15.5 (9.6–26.2)	14.3 (9.5–21.6)	<0.001^a^
MOD (units)	0.49 (0.1)	0.39 (0.13)	0.39 (0.09)	<0.001^a^
RCD (units/s)	37.8 (10.1)	41.4 (16.5)	43.4 (18.3)	0.194
T50 (min)	4.4 (0.75)	3.0 (0.7)	3.4 (0.4)	<0.001^c^

Data presented as mean (standard deviation) or median (interquartile range). *p* values by analysis of variance or the Kruskall–Wallis test. *eGFR* estimated glomerular filtration rate. Between group analysis by Tukey’s post hoc test

^a^ *p* < 0.05 between aspirin and the two other groups

^b^ *p* < 0.05 between warfarin and the two other groups

^c^ *p* < 0.05 between all three groups

Table 4 shows the same analysis of the 82 patients taking one of the three NOACs. Only 3 TEG indices differed between the NOACs. The R time (reflecting time to the initiation of clot formation) was shorter in those on apixaban than in those of dabigatran. Similarly, the TMA (time taken for the clot to be at its strongest) was shorter in those on apixaban (that being only 5.9 % longer than the time taken by those on aspirin) than in those on dabigatran (being 33.4 % longer than the time taken by those on aspirin). The CI (coagulation index, a global score of haemostasis) was higher in those on apixaban (and comparable to the CI of those on aspirin) when compared to the CI of those on dabigatran. The MPA L time (reflecting time to initiation of the clot) was shorter, and the MOD (reflecting clot density) was greater in those on apixaban and in those on rivaroxaban than in those of dabigatran. The RCF (rate of clot formation) was slower in those on rivaroxaban than in the two other NOACs. The RCD (reflecting clot dissolution by exogenous tPA) was slowest in those on rivaroxaban than in those on apixaban.

**Table 4:** Analysis according to NOAC

	Apixaban (*n* = 17)	Dabigatran (*n* = 19)	Rivaroxaban (*n* = 46)	*p*
Clinical and demographic				
Age (years)	76.9 (10.6)	73.9 (9.2)	72.1 (10.6)	0.262
Sex (male/female)	8/9	15/4	26/20	0.119
Creatinine (µmol/l)	86 (30)	90 (22)	88 (21)	0.879
eGFR (ml/min/1.73)	68 (19)	71 (14)	69 (15)	0.832
TEG indices				
R (min)	6.7 (1.6)	10.7 (5.9)	8.5 (3.2)	0.009^a^
K (min)	1.7 (0.6)	2.2 (0,9)	2.0 (0.8)	0.175
Angle (°)	64.4 (8.4)	60.9 (7.9)	63.5 (8.2)	0.371
MA (mm)	66.2 (6.5)	64.7 (5.8)	65.1 (8.1)	0.828
TMA (min)	25.0 (2.9)	31.9 (7.7)	28.5 (5.4)	0.002^a^
G (d/s/10^3^)	10.3 (2.6)	9.6 (2.6)	10.0 (3.0)	0.760
E (d/s)	205 (52)	192 (53)	199 (60)	0.760
TPI (s)	74 (45–109)	38 (30–63)	56 (40–75)	0.063
A30 (mm)	64.3 (6.6)	62.5 (5.9)	63.1 (8.1)	0.756
CL30 (%)	97.2 (2.7)	96.6 (2.2)	96.8 (2.9)	0.794
LY30	0.4 (0.05–1.1)	0.7 (0.3–2.0)	0.6 (0.1–1.3)	0.574
A60 (mm)	60.9 (6.2)	58.7 (6.2)	59.2 (7.9)	0.631
CL60 (%)	92.0 (4.2)	90.2 (3.8)	91.0 (4.5)	0.440
LY60 (%)	3.0 (1.4–4.2)	3.7 (2.0–6.1)	3.25 (1.8–4.9)	0.772
A (mm)	60.9 (6.2)	58.6 (6.2)	59.4 (7.5)	0.610
CI (arbitrary units)	2.0 (−0.8–2.8)	−2.0 (−5.0–0.1)	−0.7 (−2.5–0.7)	0.002^a^
MPA indices				
L time (min)	8.0 (7.6–9.2)	23.0 (9.8–30.3)	9.8 (7.8–12.1)	<0.001^b^
RCF (units/s)	22.0 (20.0–29.0)	28.5 (16.2–30.7)	12.4 (7.0–15.3)	<0.001^c^
MOD (units)	0.37 (0.09)	0.27 (0.09)	0.44 (0.13)	<0.001^b^
RCD (units/s)	51.8 (13.9)	42.6 (12.1)	36.8 (17.3)	0.005^d^
T50 (min)	2.9 (0.45)	3.3 (0.37)	2.9 (0.75)	0.161

Data presented as mean (standard deviation) or median (interquartile range). *p* values by analysis of variance or the Kruskall–Wallis test. *eGFR* estimated glomerular filtration rate. Between group analysis by Tukey’s post hoc test

^a^ *p* < 0.05 between apixaban and dabigatran

^b^ *p* < 0.05 between dabigatran and the two other groups

^c^ *p* < 0.05 between rivaroxaban and the two other groups

^d^ *p* < 0.05 between apixaban and rivaroxaban

## Discussion

We report marked differences in the laboratory assessment of thrombogenesis and fibrinolysis of blood and plasma from patients on different NOACs. Despite best clinical care, patients on anti-thrombotic therapy still suffer from thrombosis and are at risk of haemorrhage, as exemplified by difficulties in the management of warfarin [15–17]. Accordingly, the identification and targeted care of the highest-risk patients is likely to be successful in reducing the rate of an adverse end point, and the laboratory is best placed to provide this information.

Both the TEG and MPA methods provide data on clot formation and lysis that differ according to the anti-thrombotic drug. Seven TEG indices were different in blood taken from patients taking an OAC compared to those (on aspirin) whose coagulation pathway should be intact, and are therefore to be expected. Five indices were no different between the anti-thrombotics, calling into doubt their value in this setting. Although CL30, LY30, CL60, LY60 were different between those on warfarin and those on a NOAC, there was no difference in these indices in those on a NOAC compared to those on aspirin. Furthermore, with correlation coefficients between 0.91 and 0.99, these four indices are effectively delivering the same information. We interpret this as that the warfarin clot is less robust and faster to auto-lyse than the NOAC clot. In this respect the LY indices offer better discrimination than the CL indices. Three MPA indices reflecting time for clot formation to begin, the rate of clot formation, and clot density were understandably equally altered in oral anticoagulation use compared to aspirin. However, the T50 index was different between each of the three groups. Although the fibrin clot in those on warfarin was more susceptible to lysis by exogenous tPA (i.e. a shorter T50) than the clot in those on aspirin, the clot in those on a NOAC was significantly more susceptible to lysis.

The TEG R time and MPA L time both assess time for coagulation to begin. Compared to blood and plasma from patient on aspirin (and therefore likely to have an intact coagulation pathway), apixaban impeded initiation of thrombogenesis by an average of 37 and 45 % respectively, rivaroxaban by 75 and 78 % respectively, but dabigatran was the most effective NOAC, impeding these times by 118 to 318 % respectively. In this setting, we suggest dabigatran is the most effective NOAC. The TEG TMA and MPA RCF assess the rate of thrombogenesis. Dabigatran was least effective in interfering with thrombogenesis, inhibiting 29 % of rate of clot formation when compared to ‘normal’ thrombogenesis (i.e. when on aspirin), whilst rivaroxaban had the greatest influence of slowing thrombogenesis, giving a result (69 % inhibition) close to that of warfarin (65 % inhibition). In this respect, we suggest rivaroxaban is the most effective NOAC. The global TEG CI (coagulation index) sums several indices reflecting different aspects of thrombogenesis, including the R time, K time, MA and the angle. The result for apixaban was the same as that for aspirin, suggesting poor overall anticoagulation, whereas that for rivaroxaban was the same as for warfarin, suggesting overall equal effect. However, the increased CI figure for dabigatran suggests this NOAC has better overall function as an anticoagulant.

The MPA index MOD reflects clot density, whilst the RCD reflects the ease of digestion of the clot by tPA. Plasma from those on rivaroxaban produced the clot with the greatest density (close to that of those on aspirin) and the clot most resistant to digestion (result again close to that of aspirin). Dabigatran produced the least dense clot, whilst apixaban produced a clot that was most susceptible to thrombolysis. Despite the short T50 (reflecting rapid clot lysis) of all subjects on a NOAC compared to aspirin and warfarin users (Table 3), there was no difference between the NOACs. These data on lysis are difficult to interpret in a clinical setting. For example, it may be that a clot that is dense and resistant to lysis is more likely to promote a clinical thrombotic event, in that it is likely to remain in situ for longer, and so less likely to promote haemorrhage. Conversely, a clot that is less dense and is quicker to lyse may be less likely to transform into a clinically relevant thrombus, but may instead promote haemorrhage. In this respect, rivaroxaban produced formed clot dynamics closest to that of aspirin.

A strength of our data is that it reflects real-world oral anticoagulation use, controlled by samples from patients with the same cardiovascular disease in whom (we believe) the coagulation pathway is expected to be intact (i.e. are on aspirin). Although Dias et al. [18] probed the effects of NOACs, they spiked blood from healthy volunteers with a NOAC and subsequently applied the blood to a TEG. Neyens et al. [19] used a TEG to assess haemostasis in a case report of a patient on dabigatran. However, we must temper our conclusions in that (for clinical reasons) we recruited far fewer patients on apixaban or on dabigatran than on rivaroxaban, leading to possible false negatives in comparing apixaban with dabigatran. Furthermore, the marked correlations between many TEG indices (63 comparisons with an *r* > 0.6) suggests that a high proportion of these indices are redundant and add nothing to the value of the TEG method and, therefore, add confusion. We also acknowledge the potential effects of other medications, and of other pathology (such as inflammation and lipids) [20] that may influence fibrin clot structure (although we have excluded age, sex, body mass index and renal function as major influences). In this respect an animal model has suggested that rivaroxaban reduced levels of inflammatory cytokines [21]. A further limitation is we cannot factor in the levels of fibrinogen or the actual amount of each drug in the patient’s blood, and we were unable to determine the actual mass of each NOAC in the patient’s blood. This pertinent for the dabigatran data as active drug in the plasma may act on exogenously-added thrombin in the MPA. Nevertheless, our data point to marked differences in the extent to which selected TEG and MPA indices are influenced by warfarin and the NOACs, as are summarised in Table 5. Some of these differences are likely to reflect whole blood versus fibrin clot formation, as it is established that platelets contribute to a fibrin clot [22, 23], although our control group are taking at least 75 mg of aspirin daily, and this drug may also influence lysis by tPA [24]. Our data also point to marked differences between the effects of the NOACs, suggesting that dabigatran is a more effective agent than apixaban and in one respect, than rivaroxaban. However, one index suggests that rivaroxaban may be more effective that the other agents, whilst another index points to a weaker effect than apixaban. These variable effects cannot immediately be ascribed to the mode of action of the NOACs, that is, thrombin inhibition versus Factor Xa inhibition, and so may be specific to each NOAC.

**Table 5:** Key conclusions

Observation	Evidence	Interpretation
In whole blood and plasma, the clots formed as rapidly, and are as robust as those on a NOAC as in those on warfarin	R time, K time, MA, TMA, A30. L time, RCF, MOD	In clot formation, NOACs have an equivalent effect as warfarin
Once formed, the whole blood clot lyses at the same rate on a NOAC as on aspirin, in contrast to the rate on warfarin	LY30, LY60, CL30, CL60	The NOAC clot is quicker to lyse than the warfarin clot
The fibrin clot of those on a NOAC is more susceptible to exogenous lysis than the fibrin clot of those on warfarin	T50	NOACs provide a weaker fibrin clot than does warfarin
Dabigatran retards whole blood clot formation more effectively than does apixaban	R time, CI	Dabigatran is a more effective OAC than apixaban
As regards retarding fibrin clot formation, dabigatran is a more effective OAC than apixaban and rivaroxaban	L time TMA	Dabigatran is a more effective OAC than both other drugs
The rate of clot formation under rivaroxaban is slower than under both other NOACs	RCF	Rivaroxaban is a more effective OAC than the two other drugs
The fibrin clot formed under the effect of dabigatran is less dense than those of the other two NOACs	MOD	Dabigatran is a more effective OAC than the two other drugs
The faster rate of clot dissolution in those on apixaban than in rivaroxaban	RCD	Apixaban is a more effective OAC than rivaroxaban

*OAC* oral anticoagulant

## Conclusions

Our data suggests that the TEG and MPA methods may bring useful information in determining more precisely the mechanisms by which warfarin (as certain TEG and MPA indices correlate with the INR) and NOACs protect against thrombosis. However, the clinical value of these data is as yet unknown and can only be answered by prospective outcome studies linking these indices to clinical outcomes such as in-stent thrombosis [25].

## Abbreviations

INR: International normalised ratio
MA: Maximum amplitude
MOD: Maximum optical density
MPA: Micro-plate assay
NOAC: Non-vitamin K antagonist anticoagulant
OD: Optical density
RCD: Rate of clot dissolution
RCF: Rate of clot formation
TEG: Thrombelastograph
tPA: Tissue plasminogen activator
T50: Time for 50 % of the clot to be lysed
VKA: Vitamin K antagonist
